# Genetic basis of the association of resistance genes *mef*(I) (macrolides) and *catQ* (chloramphenicol) in streptococci

**DOI:** 10.3389/fmicb.2014.00747

**Published:** 2015-01-06

**Authors:** Marina Mingoia, Eleonora Morici, Andrea Brenciani, Eleonora Giovanetti, Pietro E. Varaldo

**Affiliations:** ^1^Unit of Microbiology, Department of Biomedical Sciences and Public Health, School of Medicine, Polytechnic University of MarcheAncona, Italy; ^2^Unit of Microbiology, Department of Life and Environmental Sciences, Polytechnic University of MarcheAncona, Italy

**Keywords:** *mef*(I), *catQ*, macrolide resistance, chloramphenicol resistance, streptococci, IQ module, integrative and conjugative elements (ICEs)

## Abstract

In streptococci *mef*(I) and *catQ*, two relatively uncommon macrolide and chloramphenicol resistance genes, respectively, are typically linked in a genetic module designated IQ module. Though variable, the module consistently encompasses, and is sometimes reduced to, a conserved ∼5.8-kb *mef*(I)-*catQ* fragment. The prototype IQ module was described in *Streptococcus pneumoniae*. IQ-like modules have subsequently been detected in *Streptococcus pyogenes* and in different species of viridans group streptococci, where *mef*(E) may be found instead of *mef*(I). Three genetic elements, one carrying the prototype IQ module from *S. pneumoniae* and two carrying different, defective IQ modules from *S. pyogenes*, have recently been characterized. All are integrative and conjugative elements (ICEs) belonging to the Tn*5253* family, and have been designated ICE*Spn*529IQ, ICE*Spy*029IQ and ICE*Spy*005IQ, respectively. ICE*Spy*029IQ and ICE*Spy*005IQ were the first Tn*5253* family ICEs to be described in *S. pyogenes*. A wealth of new information has been obtained by comparing their genetic organization, chromosomal integration, and transferability. The origin of the IQ module is unknown. The mechanism by which it spreads in streptococci is discussed.

The macrolide resistance gene *mef*(I) and the chloramphenicol resistance gene *catQ* are relatively uncommon genetic determinants accounting respectively for macrolide and chloramphenicol resistance. In streptococci, however, they are typically associated and genetically linked, since neither has yet been detected without the other. This review illustrates current data on the association and discusses its genetic basis.

## *mef* GENES

In streptococci, *mef*-class genes encode eﬄux-mediated macrolide resistance. Compared to *erm*-class genes encoding methylase-mediated target site modification, *mef* genes usually produce lower-level resistance, only affecting 14- and 15-membered macrolides (M phenotype; Sutcliffe et al., 1996). In streptococci, *mef* genes include a number of subclasses, of which *mef*(A) and *mef*(E) are the most significant. *mef*(A), the first subclass to be discovered (Clancy et al., 1996), is widespread in *Streptococcus pyogenes* but is also common in *Streptococcus pneumoniae*. *mef*(E), discovered in *S. pneumoniae* (Tait-Kamradt et al., 1997), is frequently found in this and other *Streptococcus* species, but is uncommon in *S. pyogenes* (Klaassen and Mouton, 2005). *mef*(I), discovered in *S. pneumoniae* (Cochetti et al., 2005; accession no. AJ971089) and subsequently detected in *S. pyogenes* (Blackman Northwood et al., 2009) and viridans group streptococci (VGS; Brenciani et al., 2014), is the most widely investigated among the other *mef* subclasses; these include *mef*(O), *mef*(B), and *mef*(G), detected respectively in *S. pyogenes* (Sangvik et al., 2005), *Streptococcus agalactiae* (Cai et al., 2007), and group G streptococci (Amezaga and McKenzie, 2006; Cai et al., 2007). An *msr*-class gene with homology to *msr*(A), involved in macrolide eﬄux in *Staphylococcus aureus* (Ross et al., 1990), is located immediately downstream of the *mef* gene: it is usually designated *msr*(D), although a number of variants are associated with different *mef* genes. Cotranscription of *mef*(E) and *msr*(D) in *S. pneumoniae* suggested that the products of the two genes may act as a dual eﬄux system (Gay and Stephens, 2001).

## *cat* GENES

The first and still predominant mechanism of bacterial resistance to chloramphenicol is enzymatic inactivation of the drug by different chloramphenicol acetyltransferases (CATs) encoded by *cat* genes. Two types of CATs have been recognized, A and B, each including a number of structurally and phylogenetically diverse groups (Schwarz et al., 2004). The *catQ* gene, encoding a type-A CAT assigned to a distinct group (Schwarz et al., 2004), was originally described (Rood et al., 1989) and then sequenced (Bannam and Rood, 1991; accession no. M55620) in *Clostridium perfringens* CW531. However, no further *catQ* gene from this species has been described or is available in GenBank, even though genes with 77–92% DNA identities to *catQ* from *C. perfringens* CW531 can be found in several other *Clostridium* species by *in silico* analysis. Different type-A CATs encoded by a number of *cat* genes have been described in streptococci (Trieu-Cuot et al., 1993; Schwarz et al., 2004). With regard to *catQ*, an early study reported a *catQ*-like gene in a *S. agalactiae* strain (Trieu-Cuot et al., 1993). More recently, Mingoia et al. (2007) described *catQ* in two *S. pneumoniae* isolates and found that the *catQ* gene from *S. pneumoniae* Spn529 (the reference isolate) displayed 96.2% DNA identity (accession no. AJ971089) to the original *catQ* from *C. perfringens* CW531.

## THE IQ MODULE

**FIGURE 1:**
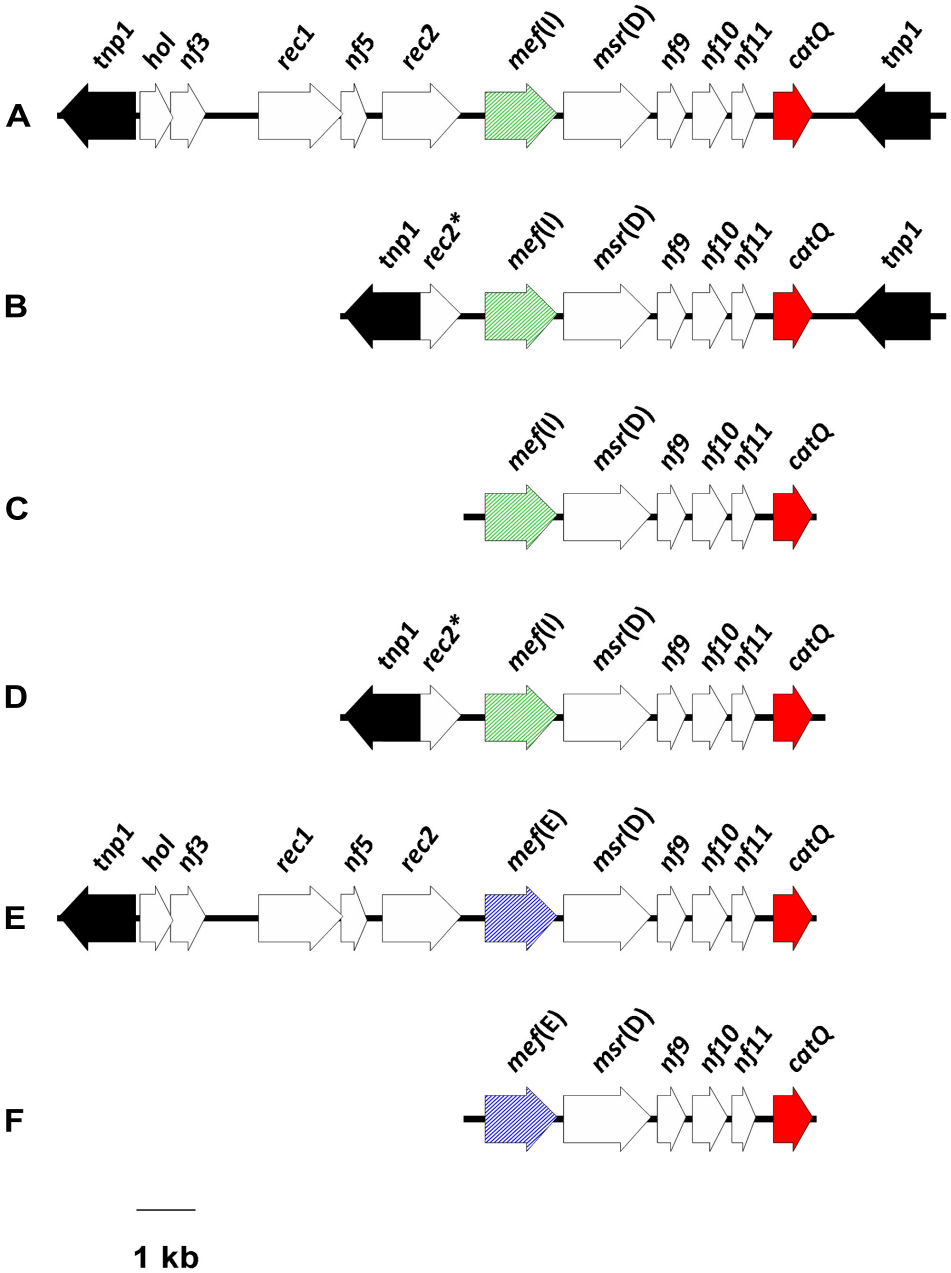
**Schematic illustration of the IQ modules detected so far in *Streptococcus* species. (A)** *S. pneumoniae*; prototype IQ module (Mingoia et al., 2007); **(B)** *S. pyogenes* (Del Grosso et al., 2011); **(C)** *S. pyogenes* (Del Grosso et al., 2011); **(D)** *S. mitis* (Brenciani et al., 2014); **(E)** *S. oralis* (Brenciani et al., 2014); **(F)** *S. mitis*, *S. sanguinis*, *S. parasanguinis* (Brenciani et al., 2014). The ORFs are depicted as arrows pointing in the direction of transcription: the *mef* gene is diagonally striped (green, *mef*(I); blue, *mef*(E)); *catQ* is red; the transposase gene *tnp1* is black; and the other ORFs are white.

When *catQ* was first detected in *S. pneumoniae*, it was found to be linked to *mef*(I) in an ∼15.1-kb DNA fragment, containing two identical *tnp1* transposase genes at either end, that was designated ‘IQ element’ (Mingoia et al., 2007). Here, we prefer to call it ‘IQ module,’ since the term ‘element’ might be confusing. Two defective IQ modules were subsequently described in *S. pyogenes*: one was characterized by a shorter region upstream of *mef*(I), formed only by *tnp1* and a truncated recombinase gene *rec2*; the other consisted of the sole *mef*(I)-*catQ* fragment (Del Grosso et al., 2011). Very recently, a number of variously defective IQ-like modules have been detected in different VGS species (Brenciani et al., 2014). An interesting characteristic of VGS modules is that they most often bear *mef*(E) instead of *mef*(I). Moreover, all IQ-like modules detected in VGS lack the right *tnp1*, i.e., the transposase gene downstream of *catQ* that is found in the IQ module of *S. pneumoniae*. Despite variability upstream of the *mef* gene and downstream of *catQ*, all reported IQ modules appear to share a conserved *mef*-*catQ* fragment [six open reading frames (ORFs), ∼5.8 kb; **Figure 1**].

The origin of the IQ module is unknown. The prototype IQ module of pneumococcal origin has been proved to be capable of undergoing excision in circular form, but was unable to be transferred to a recipient (Mingoia et al., 2014).

## FROM THE 5216IQ COMPLEX TO A Tn*5253* FAMILY ICE

The first attempt to clarify the genetic basis of the *mef*(I)-*catQ* association dates back to 2007, when Mingoia et al. (2007) identified in *S. pneumoniae* a composite structure, that they designated 5216IQ complex (∼30.5 kb); it consisted of two moieties, one represented by the IQ module and the other formed by fragments of transposons Tn*5252* and Tn*916*. The Tn*5252* fragment corresponds to the conjugal transfer-related (CTR) functional module of Tn*5252* (Alarcon-Chaidez et al., 1997); the Tn*916* fragment contains a silent *tet*(M), unexpressed because it lacks the promoter, the ribosome-binding site, and part of the leader peptide. As realized later (Mingoia et al., 2014) also thanks to the new notion of integrative and conjugative element (ICE; Seth-Smith and Croucher, 2009; Wozniak and Waldor, 2010), the so-called 5216IQ complex is not in fact a genetic element (Varaldo et al., 2009), but rather part of a larger element, a Tn*5253* family ICE.

## GENETIC ELEMENTS CARRYING IQ MODULES

The genetic location of the IQ module has recently been investigated in three strains: one *S. pneumoniae* (Spn529, serotype 11A [Mingoia 07]) and two *S. pyogenes* (Spy029 and Spy005, respectively *emm* types 25.0 and 12.42 [Del Grosso 11]). All three IQ modules were found to be inserted in Tn*5253* family ICEs, which were respectively designated ICE*Spn*529IQ, ICE*Spy*029IQ, and ICE*Spy*005IQ (Mingoia et al., 2014). It is worth noting that this was the first time that Tn*5253* family ICEs were described in natural isolates of *S. pyogenes*. The family prototype, Tn*5253*, is a long-established conjugative composite element resulting from the insertion of Tn*5251* (virtually identical to Tn*916*) into Tn*5252* (Ayoubi et al., 1991). Several studies have recently addressed the heterogeneity of Tn*5253*-like composite elements (Croucher et al., 2009, 2011; Ding et al., 2009; Henderson-Begg et al., 2009; Mingoia et al., 2011; Wyres et al., 2013), whose integrase genes may belong to one of two types: *int_5252_* (the gene of the Tn*5253* prototype) or *int_Sp_*_23FST81_ [first described in ICE*Sp*23FST81 (Croucher et al., 2009)]. The complete sequence of Tn*5253* is available (accession no. EU351020) and has been extensively analyzed (Iannelli et al., 2014).

**FIGURE 2:**
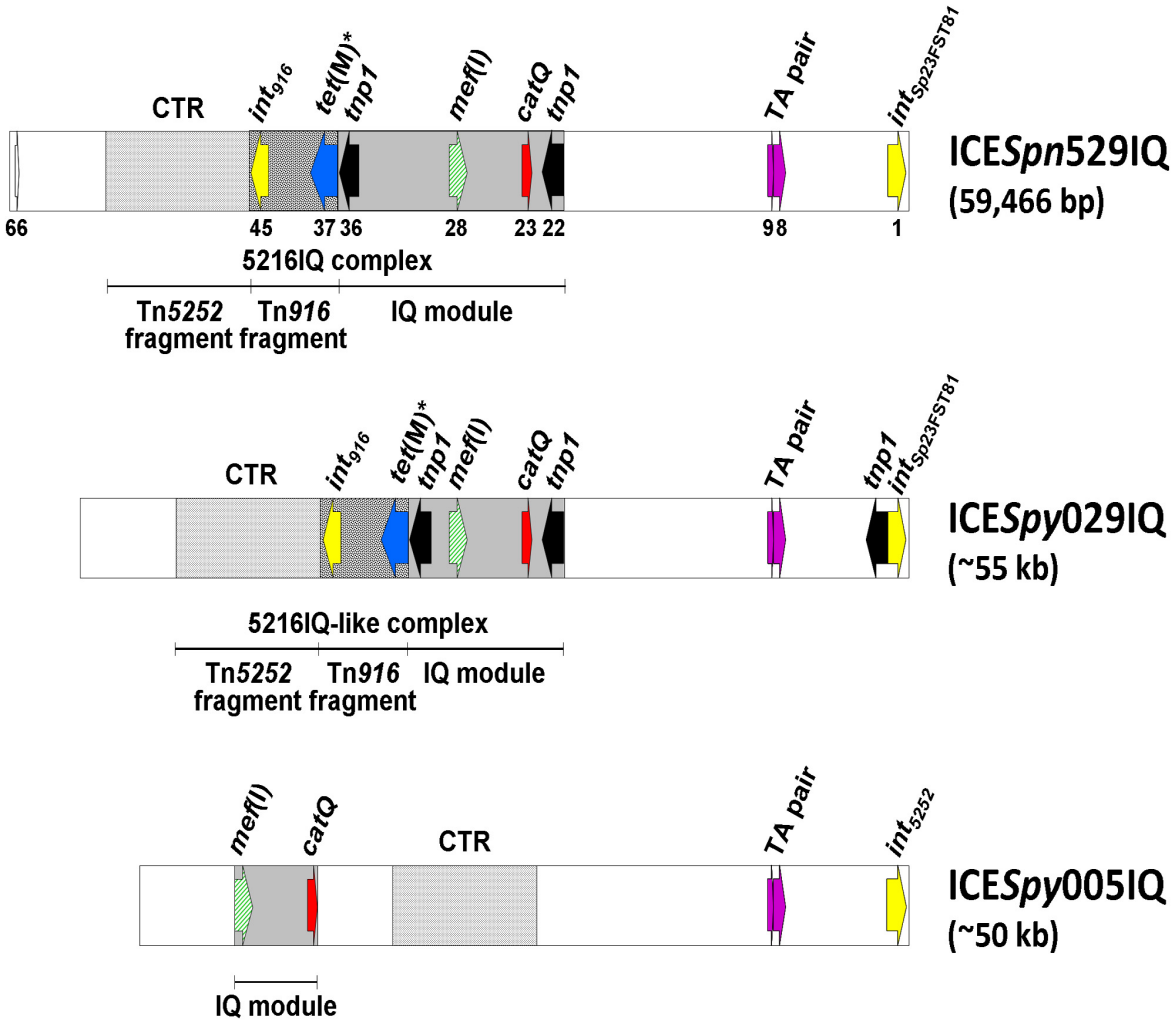
**Schematic representation of ICE*Spn*529IQ, completely sequenced, and of ICE*Spy*029IQ and ICE*Spy*005IQ, characterized by PCR mapping, partial DNA sequencing and restriction analysis.** The ICE genomes are represented as white rectangles with some ORFs and some modules or fragments in evidence. The IQ module is depicted as a gray rectangle, the Tn*916* fragment as a spotted rectangle, and the CTR module as a dotted rectangle. The ORFs are depicted as arrows pointing in the direction of transcription, including *mef*(I) (diagonally striped green), *catQ* (red), *tnp1* (black), integrase genes (yellow), TA gene pair (purple), and silent *tet*(M) (blue). *orf66* of ICE*Spn*529IQ is white. The progressive number is reported only below selected ones of the 66 ORFs of ICE*Spn*529IQ.

ICE*Spn*529IQ has been completely sequenced (accession no. HG965092): it is 59,466 bp long; its G+C content is 36%; and sequence analysis disclosed 66 ORFs. The IQ module spans from *orf22* to *orf36* (which correspond to the two identical *tnp1* transposase genes located at either end of the module); *catQ* is *orf23* and *mef*(I) is *orf28*. ICE*Spy*029IQ and ICE*Spy*005IQ have been characterized based on PCR mapping, partial DNA sequencing, and restriction analysis. The three ICEs are shown and compared in **Figure 2**.

ICE*Spn*529IQ and ICE*Spy*029IQ appear to share a very similar organization. Both carry the integrase gene *int_Sp_*_23FST81_ and an identical Tn*916* fragment. Aside from the different size of their IQ modules, which nevertheless share the same position and insertion site in the respective ICE, the only noticeable difference between the two ICEs is a transposase gene *tnp1* adjacent to *int_Sp_*_23FST81_; as a result ICE*Spy*029IQ carries a third *tnp1* in addition to those found at the ends of the IQ module. In contrast, ICE*Spy*005IQ has the integrase gene *int_5252_* and no Tn*916* fragment, and its defective IQ module is found in a different position compared to the IQ modules of ICE*Spn*529IQ and ICE*Spy*029IQ.

All three ICEs lack the linearized plasmid pC194, which contains the chloramphenicol resistance determinant *cat*_pC194_ and is usually a distinctive cargo of the Tn*5252*-like moiety of Tn*5253* family ICEs. This might be a fitness-related characteristic: as if the ICE no longer needed the plasmid since chloramphenicol resistance is already assured by *catQ*. It is well known since early studies that the DNA region containing the linearized pC194 is flanked by direct repeats whose recombination may lead to spontaneous curing of the region (Ayoubi et al., 1991; Kiliç et al., 1994). Recent analysis of the sequence of Tn*5253* (Iannelli et al., 2014) disclosed a 7,627-bp Ω*cat*(pC194) flanked by two 1,169-bp direct repeats, which contain a toxin-antitoxin gene pair (Palmieri et al., 2013) that has previously been detected in the pathogenicity island PPI-1 of *S. pneumoniae* strain TIGR4 (Khoo et al., 2007). In all three ICEs the lack of the integrated plasmid pC194 is associated with the presence in the corresponding region of a single copy of this toxin-antitoxin operon (TA pair), which likely represents the recombination site of Ω*cat*(pC194) and might be the outcome of repair after plasmid excision. Of the three ICEs, only ICE*Spy*005IQ has been detected as a free circular form, a critical condition for an ICE to undergo conjugal transfer (Wozniak and Waldor, 2010).

## TRANSFERABILITY OF *mef*(I) AND *catQ*

The transferability of *mef*(I) and *catQ* varies with the transfer mechanism (conjugation or transformation) and the strain harboring the IQ module. Using *S. pneumoniae* donors, transfer of *mef*(I) and *catQ* has never been obtained to any recipient by conjugation, whereas *mef*(I)- and *catQ*-positive transformants have been obtained by transformation (Nielsen et al., 2010; Mingoia et al., 2014). It is worth noting that, in experiments using *S. pneumoniae* Spn529 as the donor, transformants were obtained at a very low frequency (around 10^-9^) and only using a crude lysate as the transforming DNA: this suggests that DNA may undergo changes during the extraction process, which may account for the early negative results (Mingoia et al., 2007). Conversely, using *S. pyogenes* donors, conjugation assays yielded no transconjugants from Spy029, whereas transconjugants harboring the entire ICE*Spy*005IQ were obtained at high frequencies from Spy005 (Del Grosso et al., 2011; Mingoia et al., 2014). Transformants were obtained with both *S. pyogenes* donors, but at a high frequency from strain Spy005 and at a very low frequency from strain Spy029.

## SPREAD OF *mef*(I) AND *catQ* IN STREPTOCOCCI

It has been argued by analogy with other *mef* gene subclasses (especially in pneumococcal populations) that the spread of *mef*(I) — and of the IQ module, where it is consistently found — could largely depend on transformation mechanisms (Mingoia et al., 2014). In *S. pneumoniae*, the two major *mef* determinants, *mef*(E) and *mef*(A), are carried respectively by the mega element (Gay and Stephens, 2001) and Tn*1207.1* (Santagati et al., 2000), whose relationship with the relevant *mef* gene is roughly comparable to that of the IQ module with *mef*(I). Similar to the IQ module, the mega element and Tn*1207.1* are transferable by transformation rather than by conjugation. While the mega element has a variety of insertion sites in the pneumococcal chromosome and its dissemination is erratic, Tn*1207.1* is integrated into the chromosome at a specific site and its dissemination is mainly clonal (Del Grosso et al., 2002). Non-clonal dissemination of the IQ module is suggested by the wide range of serotypes of the *S. pneumoniae* isolates that have so far been found to be *mef*(I)-positive (Cochetti et al., 2005; Reinert et al., 2008; Nielsen et al., 2010); moreover *S. pyogenes* strains Spy029 and Spy005 also belong to different *emm* types (Del Grosso et al., 2011). Altogether, these data suggest that the behavior of *mef*(I) is likely to be more similar to that of *mef*(E) than of *mef*(A).

## CONCLUDING REMARKS

In streptococci, the macrolide resistance determinant *mef*(I) and the chloramphenicol resistance determinant *catQ*, both relatively uncommon within the respective class of resistance genes, are typically linked in a genetic module, designated IQ module. Although the IQ module is uncommon in streptococci, it is likely to be unique to them: it has not been reported outside the genus *Streptococcus*, and its origin is unknown. Though variable, the IQ module consistently encompasses, and may be reduced to, a conserved ∼5.8-kb DNA fragment spanning from *mef*(I) (or *mef*(E) in some VGS) to *catQ*. After early report in *S. pneumoniae* (Mingoia et al., 2007), IQ-like modules have been detected in *S. pyogenes* (Del Grosso et al., 2011) and VGS (Brenciani et al., 2014); moreover, Mingoia et al. (2014) have recently reported on ongoing studies of isolates of other *Streptococcus* species (*S. agalactiae*) harboring linked *mef*(I) and *catQ* genes.

In recent years, neither *mef*(I) nor *catQ* have been described in the documented absence of the other in streptococci. However, the association is unlikely to have been the rule in earlier investigations. Indeed, the *S. agalactiae* strain where a *catQ*-like gene was first described in a streptococcus was erythromycin susceptible (Trieu-Cuot et al., 1993). The same is probably true of clostridia: *C. perfringens* CW531, where *catQ* was originally detected and sequenced even though its genetic environment was not investigated, was reported to be resistant, besides chloramphenicol, to tetracycline, but not to erythromycin (Rood et al., 1989); and there is no evidence of associated *mef* genes in the sequenced clostridial genomes or contigs containing *catQ*-related genes.

## Conflict of Interest Statement

The authors declare that the research was conducted in the absence of any commercial or financial relationships that could be construed as a potential conflict of interest.
